# The Spatial Allocation of Hospitals With Negative Pressure Isolation Rooms in Korea: Are We Prepared for New Outbreaks?

**DOI:** 10.34172/ijhpm.2020.118

**Published:** 2020-07-08

**Authors:** Hyoungah Kim, Dohyeong Kim, Christopher Paul, Chang Kil Lee

**Affiliations:** ^1^Institute of Public Policy and Administration, Chung-Ang University, Seoul, South Korea.; ^2^School of Economic, Political and Policy Sciences, University of Texas at Dallas, Richardson TX, USA.; ^3^Department of Public Administration, North Carolina Central University, Durham, NC, USA.; ^4^Department of Urban Policy and Administration, Incheon National University, Incheon, South Korea.

**Keywords:** Infectious Disease Outbreak, Health Facility Allocation, Geographic Information System, Evidence-Based Health Policy, South Korea

## Abstract

**Background:** Allocation of adequate healthcare facilities is one of the most important factors that public health policymakers consider when preparing for infectious disease outbreaks. Negative pressure isolation rooms (NPIRs) are one of the critical resources for control of infectious respiratory diseases, such as the novel coronavirus disease 2019 (COVID-19) outbreak. However, there is insufficient attention to efficient allocation of NPIR-equipped hospitals.

**Methods:** We aim to explore any insufficiency and spatial disparity of NPIRs in South Korea in response to infectious disease outbreaks based on a simple analytic approach. We examined the history of installing NPIRs in South Korea between the severe acute respiratory syndrome (SARS) outbreak in 2003 and the Middle East respiratory syndrome coronavirus (MERS-Cov) in 2015 to evaluate the allocation process and spatial distribution of NPIRs across the country. Then, for two types of infectious diseases (a highly contagious disease like COVID-19 vs. a hospital-based transmission like MERS-Cov), we estimated the level of disparity between NPIR capacity and demand at the sub-regional level in South Korea by applying the two-step floating catchment area (2SFCA) method.

**Results:** Geospatial information system (GIS) mapping reveals a substantial shortage and misallocation of NPIRs, indicating that the Korean government should consider a simple but evidence-based spatial method to identify the areas that need NPIRs most and allocate funds wisely. The 2SFCA method suggests that, despite the recent addition of NPIRs across the country, there should still be more NPIRs regardless of the spread pattern of the disease. It also illustrates high levels of regional disparity in allocation of those facilities in preparation for an infectious disease, due to the lack of evidence-based approach.

**Conclusion:** These findings highlight the importance of evidence-based decision-making processes in allocating public health facilities, as misallocation of facilities could impede the responsiveness of the public health system during an epidemic. This study provides some evidence to be used to allocate the resources for NPIRs, the urgency of which is heightened in the face of rapidly evolving threats from the novel COVID-19 outbreak.

## Background

Preparation and response for infectious disease outbreaks is one of the most important roles for public health authorities. The increasing emergence of virulent infectious diseases due to globalization and environmental changes is further cause for concern,^1^ as is evidenced in the coronavirus disease 2019 (COVID-19) outbreak originating in Wuhan, China and leading to a global pandemic in 2020.^2^ Prior to the COVID-19 pandemic, which is still ongoing at the time of this study, South Korea had experienced three notable infectious disease outbreaks in recent history: severe acute respiratory syndrome (SARS) in 2003, H1N1 (influenza A) in 2009, and Middle East respiratory syndrome coronavirus (MERS-CoV) in 2015.^3^ In 2015, the MERS-CoV became an outbreak of concern because of the uncertainty of presentation and management across countries.^4^ The determinants of public health and socioeconomic outcomes of MERS-CoV have been extensively explored and investigated by the global community.^5-7^ In the regional comparison study of MERS-CoV, the strength of the public health system and effectiveness of outbreak response is one of the critical factors in variation of MERS-CoV outcomes.^1^ In an infectious disease outbreak, central or regional government agencies implement a variety of response policies and programs such as immunization, screening, and quarantine in order to limit the spread of disease.^8^ Among these policies, preparing sufficient numbers of infection control facilities in hospitals is a key factor to reduce disease transmission in a country.^9,10^

For infectious respiratory diseases, one key resource for infection control are negative pressure isolation rooms (NPIRs). NPIRs prevent the transmission of airborne infection by lowering air pressure in the isolation room relative to hospital corridors or other rooms.^9,11^ If NPIRs are built with several isolation functions (such as negative pressure under continuous electronic monitoring, HEPA-filtered air, and separate bathrooms and anterooms), the possibility of contagion of respiratory infectious diseases become almost zero. The cost of NPIRs, however, is quite high. Private hospitals hesitate to build them because the financial returns of new NPIR facilities are not beneficial.^12^ Due to the high-cost and relatively low-returns, it is argued that NPIRs are public goods that governments should provide to increase the level of preparation for virulent infectious disease outbreaks.^10^

Predicting and forecasting infectious disease are very difficult problems in public health planning due to the many complex factors affecting disease spread, notably human behavior.^13,14^ In one recent incident in the COVID-19 outbreak in South Korea, a religious gathering was a catalyst of dramatic increase of confirmed cases (they represented almost 80% of confirmed cases at the end of February, 2020). If the first step for preparation of epidemic is surveillance, then the second step is to mitigate rapid transmission through the timely isolation and treatment of confirmed cases to prevent community-acquired infection.^15^ This requires preparedness and appropriate allocation of healthcare facilities. A government, therefore, should consider the allocation of health facilities in which confirmed patients can be promptly isolated and treated. Allocation decisions should be made by local population density and facility capacity in pessimistic scenarios.

This study proposes that a simple spatial approach to allocation can highlight gaps in preparation. The goal of this study is to evaluate the allocation process and spatial distribution of NPIR hospitals across South Korea since the year of 2000 and the implications for future allocation. The paper proposes evidence-based spatial allocation methods which can indicate specific locations where the central government’s funds should be allocated to install more NPIRs. Using the case of 2015 MERS-CoV outbreak, the paper models the specific sites of need in South Korea, the urgency of which is heightened in the face of threats from the novel COVID-19 outbreak. In addition to facility allocation, of course, many other factors determine the effectiveness of outbreak response. In this context, South Korea is a good research subject because it has experienced several infectious disease outbreaks, one of which the country successfully contained (SARS) and others of which it was largely unsuccessful (MERS) in containing. Through the analysis of historical experiences of SARS, H1N1, and MERS, we scrutinize the chronological process of NPIR allocation in South Korea and evaluate the impact of health resource misallocation. Our study warns of NPIR allocation driven by political convenience and suggests a simple approach to evidence-based analysis with limited data.

### 2015 MERS-CoV Outbreak in South Korea

Prior to the COVID-19 pandemic, MERS-CoV was the most severe outbreak in Korea in recent history. Over 12 000 people were isolated for MERS-CoV, and 38 deaths out of 186 confirmed cases were attributed to the disease (representing a mortality rate of 20.4%).^16^ The number of MERS-CoV cases in South Korea surpassed every other country in the world except for Saudi Arabia. Furthermore, the outbreak had a significant economic impact on the country decreasing the economic growth rate by 0.5 percentage points, representing a $10 billion loss.^17^

Nearly all MERS-CoV infections in South Korea were linked to exposure in hospitals which hosted suspected or confirmed MERS cases.^4,18^ Forty-four percent of confirmed cases were admitted or treated in the same hospital with a confirmed case, 38% of them were either family members or visitors, and 17% were medical staff.^19^ Thus, a key concern arising from the tragic MERS outbreak was the lack of special healthcare facilities such as NPIRs.^7,19^

Although the importance of NPIRs has been revived during the COVID-19 outbreak,^20^ there has been no scientific study that assesses the adequacy of spatial allocation of NPIRs in South Korea. In 2003, Taiwan had a total of 764 NPIRs when experiencing SARS. Taiwanese NPIR facilities were appropriately equipped with HEPA-filtered air, negative pressure under continuous electronic monitoring, separate bathrooms, and so on. In the outbreak, The Taiwan Center for Disease Control ensured the first 23 possible patients were treated and cared in these NPIRs in 15 hospitals as successfully prevented transmission unlike other nearby countries where NPIRs were not commonly available in hospitals.^21^ Also, a study on the allocation of NPIRs in Japan indicated the need to increase the number of NPIRs because nurses in hospitals without NPIRs were more likely to be exposed to latent tuberculosis infection after the 2011 Great East Japan Earthquake.^22^

The primary recommendation of the White Report,^23^ published after the MERS-CoV outbreak by the Korean Ministry of Health and Welfare, was to increase the number of isolation health facilities like NPIRs. Indeed, many studies have concluded that the Korean government should prepare more effective isolated health facilities across the country following the shortages revealed by MERS-CoV.^7,19,24^ Although systematic planning of isolation health facilities may not generate immediate benefit due to uncertain characteristics of novel contagious outbreaks, it is worthwhile to assess the existing distribution of such facilities nationwide and identify underprovided areas needing policy attention.

### Evidence-Based Health Resource Allocation

Health resource allocation is a key administrative decision for governments, which would suggest that institutional structures should guide decision-making through established processes. Yet, public policy decisions in fact occur in the context of uncertainty in multiple streams of problems, policy, and politics.^25,26^ Political power structures are often a primary factor in policy-making^27,28^ and decision-making for health resource allocations are frequently determined by administrative convenience and political dynamics.^29^ In contrast to the uncertainties of politics, evidence-based decision-making methods employ a systematic decision-making approach, utilizing technology and information to optimize choices. Evidence-based decision-making can improve equity in resource allocation and health facility accessibility by relying on measurable knowledge, rather than political power structures, to determine in health resource allocation. As a result, this approach can increase the level of social welfare and the degree of health equity.^18,27^ Moreover, decision-makers can be held accountable for health facility allocations, which can increase public acceptance of resource allocation decisions.^27^ The World Health Organization (WHO) guidance for health facility allocation promotes this structured process^30^ and many developed countries have begun to adopt the evidence-based methods in public health facility allocation and in health policy decision-making.^26,31^

Despite broad benefits, in practice, on-going political and economic pressures prevent many countries from successfully incorporating evidence-based facility allocation models. When a country experiences an outbreak of an infectious disease, the government may consider building special healthcare facilities. In such an emergency, political dynamics often lead to instant decisions to allocate or establish additional healthcare facilities. Because these decisions are not carefully weighed in a broader context, they often increase inequality in resource allocation among hospitals, and worsen equity of access to healthcare. Particularly in developing countries, politicized health allocation has led to facility numbers and spatial allocations to be inefficient and intensify existing inequalities.^32^ Many countries, including South Korea, have found it difficult to adopt the evidence-based methods for health facility allocation.^26^

### Allocation Process for NPIR Funds in South Korea

In South Korea, allocating healthcare resources is the focus of substantial public policy attention, particularly around perceived risks from emerging infectious diseases such as coronaviruses. However, national allocation has not been conducted with optimization models, but rather steered by political factors, local resources, or administrative convenience. NPIRs are a specific healthcare resource identified in the national plan of South Korea.^33^ The 2015 national plan targeted the number of a single room NPIRs to increase by nearly 100 units. In 2016, 19 hospitals across the country had a total of 119 NPIR beds, and the central government of South Korea committed to subsidizing 23.4 billion won (equivalent to US$22 million) per NPIR that a hospital newly builds.

**Figure 1:**
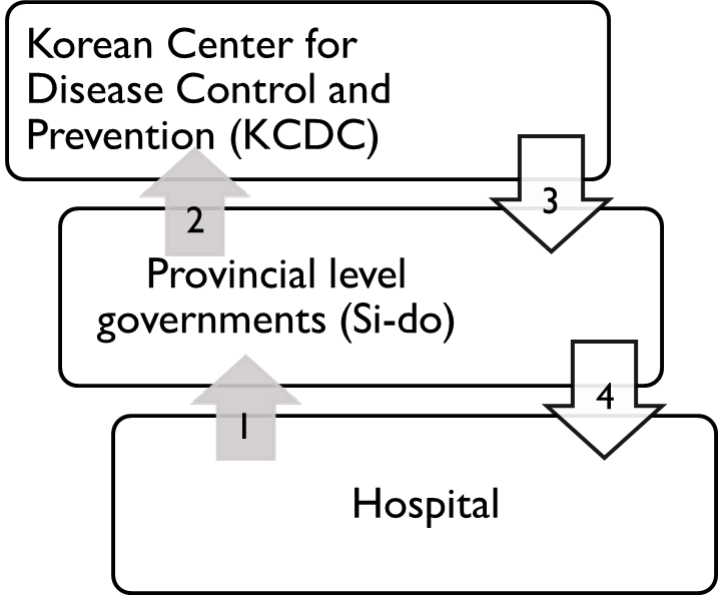
The Process of NPIR Resource Allocation in South Korea. Abbreviation: NPIR, negative pressure isolation room.

The national plan for NPIR creation in South Korea details the process of subsidy award for NPIR construction.^33^ Hospitals with existing NPIRs have priority for receiving the subsidies, but other hospitals may apply only if they meet specific regulation requirements pertaining to: size, type, number of hospital medical staff, other ancillary facilities in the hospital, and infectious disease experience. Once a hospital meeting the aforementioned requirements applies for NPIR grants, local governments of the hospitals review these applications, attach recommendations, and send these applications to a central government agency, the Korean Center for Disease Control (KCDC). When an application is selected by the KCDC, funding and other resources are granted to local governments, which distribute them to the hospitals. Figure 1 depicts this process.

Although this decision-making process is efficient and avoids many social conflicts, it is likely to result in unequal resource distribution. First, the resource allocation process is a merit-based system. Because wealthy hospitals often have better healthcare outcomes, and human and material resources, they also have a higher chance of receiving NPIR funding, exacerbating the problem of unbalanced resource distribution. Second, while the current process is convenient for decision-makers, it gives them no opportunity to consider the outcomes of an allocation or test the implications of their decisions. Finally, the direct beneficiaries of NPIRs are not considered in the resource allocation process. The current process assumes that hospitals with better facilities will have more respiratory disease patients. Accordingly, large hospitals, able to satisfy all other requirements, have better chances to obtain NPIR resources. Within this context, comparing spatial distribution of NPIRs with those of MERS-CoV patients and population size can reveal a potential mismatch between supply and demand of public health resources and highlight the importance of evidence-based decision-making processes to public health policy.

## Methods

### Data

The demographic data including populations of the years of 2009 and 2015 for South Korea were obtained from the Korea Statistical Information Service website (http://kosis.kr/eng). The infectious disease patient and hospital data are open source and available in the disease web statistics system organized by the KCDC. Other geospatial information system (GIS) related data, including shapefiles for Korea Topographic Map, were available on ArcGIS websites(http://www.arcgis.com/home/item.html?id=e220cddfd0104a20b49a05da1a89aece). The data are aggregated at the level of 250 municipalities in South Korea (*si-gun-gu*), and each of these municipalities serve as a unit of analysis of this study. The patient records and population data for both influenza A (2009) and MERS-CoV (2015) were used for creating maps of the historical changes of patients and NPIR allocations. The SARS patient data were excluded because of very small number of cases (n = 3) and no mortality from the disease in South Korea. However, the SARS outbreak brought awareness of NPIR facilities for the Korean government and made it start to install NPIRs in major hospitals. Table shows the descriptive information of population size and number of Influenza A and MERS-CoV cases by municipalities in South Korea.

**Table:** Descriptive Statistics of Each Dataset

**Dataset**	**Mean**	**SD**	**Min**	**Max**	**N**
2009 Population size by municipalities	198 753	155 587	10 168	867 678	250
2015 Population size by municipalities	206 117	161 973	10 153	848 987	250
Number of influenza A cases by municipalities (2009)	2806	2673	48	16 272	250
Number of MERS-CoV cases by municipalities (2015)	0.73	2.48	0	29	250

Abbreviations: SD, standard deviation; MERS-CoV, Middle East respiratory syndrome coronavirus.

### Analytic Methods

To explore and diagnose the current allocation of NPIRs in South Korea, we used a GIS approach to assess allocations, coverage, and disparities of NPIR facilities. First, a series of mapping (chronological GIS mapping) was conducted to illustrate the chronological variation of NPIR allocation throughout major infectious disease outbreaks since 2003 when South Korea experienced SARS. In this analysis, we discover the role of political factors in non-optimized allocation of health resources, creating a mismatch between health facilities and needs. Second, we used a two-step floating catchment area (2SFCA) method to measure recent NPIR coverage for different outbreak scenarios, based on patient records and population data. The 2SFCA method permits estimation of the level of disparity between NPIR capacity and confirmed MERS cases or population distribution. This analysis has the advantage of measuring the coverage of facilities using buffers, taking into account healthcare accessibility and spatial decomposition.^34^ The basic 2SFCA method consists of two steps assessing healthcare access: supply and then demand of health facilities. In the first step, a set of travel time from a health service provider, calculated by road distance and average driving speed, is used to aggregate the population that a supplier can reach. Then, a provider-to-population ratio is computed. Thus, if the ratio is 1 then the total population can reach the healthcare provider within the given driving distance. The second step is summing all provider-to-population ratios of health facilities within a certain driving distance from a particular population center, which we defined as spatial accessibility index. The mathematical expression for these is as follows. In the result maps, the values of $A_{i}^{F}$ are depicted.

Step 1: $R_{j}=\frac{S_{j}}{\sum_{k\in Dis\tan ce\leq do}^{n}P_{k}}$

Step 2: $A_{i}^{F}=\sum R_{j}$

Where:

*S*_j_: Medical capacity at each provider *j*

*R*_j_: Provider-to-population ratio at each provider *j*

*P*_k_: Population (or demand) site *k*

*d*_o_: Travel threshold

Distance_k,j_: Travel time between *k* and *j*

$A_{i}^{F}$: Spatial accessibility index of each population (or demand) site *i*

This method has the advantage of producing results that are easily understood and interpreted, using a relatively simple method with limited data. The buffer zones visually show the boundaries of each health facility’s capacity, considering the interaction between supply and demand. Our approach uses a simple method of 2SFCA rather than an enhanced one because of data limitations and capacity constraints facing infectious disease personnel. The method has some disadvantages, including the assumption that it is equally easy to access all providers within the designated drive time, not taking account of traffic. Travel time in urban and rural areas are also assumed to be the same. Furthermore, the method is concerned with only one type of transportation. Moreover, in an outbreak, medical personnel can transfer to hospitals where infected cases are being treated. This study uses only bed numbers in NPIRs to illustrate how insufficient NPIR resources are across the country in preparedness for the next infectious disease outbreak. While more complex versions of 2SFCA exist,^35,36^ these are not needed to conclude there are insufficient and poorly allocated NPIRs. The application of a simple method with easily available data conveys the value of simple analytical approaches for baseline assessments in low resource settings, which has been known to enhance the level of policy adoption and implementation.^37,38^ With the 2SFCA method, we also conducted a sensitivity analysis of the need for NPIRs in pessimistic and optimistic scenarios based on the travel distance of suspected patients from 10 miles to 40 miles (roughly, one-hour driving). We also evaluated the conditions of two different types of infectious diseases; MERS-like hospital-based transmission (using the reported MERS case data in 2015) vs. highly contagious disease like COVID-19 occurring through social interactions (suggesting COVID-19 demand for NPIRs will be in equal proportion for the population).

## Results

### Descriptive Analysis and Mapping

**Figure 2:**
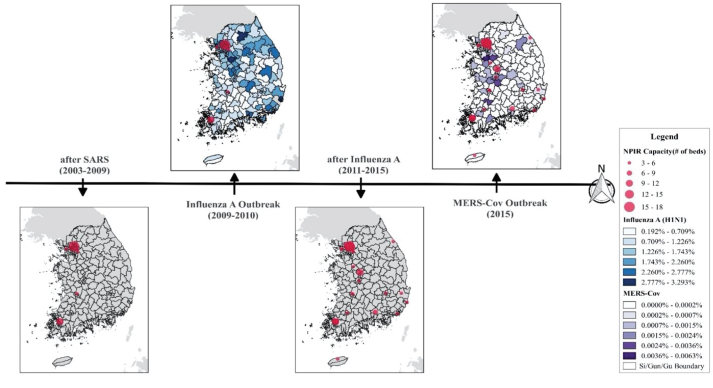
Chronological Allocation Changes of NPIR Hospitals in South Korea From 2003 to 2015. Abbreviations: NPIR, negative pressure isolation room; MERS-CoV, Middle East respiratory syndrome coronavirus; SARS, severe acute respiratory syndrome.

Figure 2 illustrates the changing location of NPIRs over time in South Korea, from the SARS outbreak in 2003 to the MERS outbreak in 2015. As shown, there were no confirmed SARS deaths, but the SARS outbreak brought awareness of NPIR facilities in the country for the first time, and the Korean government began to install NPIRs in major hospitals across the country.^39^ However, the location of NPIRs was unbalanced, and disproportionately concentrated in the western areas. Most resources were clustered around the Seoul metropolis, the capital of the country and home to one fourth of the national population. The size of the red circles in Figure 2 indicates the capacity of NPIRs. Meanwhile, two other areas also received funds for NPIR facilities, but government documents give no explanation of how these places were chosen. After the influenza A (H1N1) outbreak in 2009, the Korean government increased the number of NPIRs, as shown in the third map in Figure 2. Some hospitals in the west increased NPIR capacity, and new NPIRs were built in eastern areas, resulting in NPIRs being more evenly spread throughout the country.

**Figure 3:**
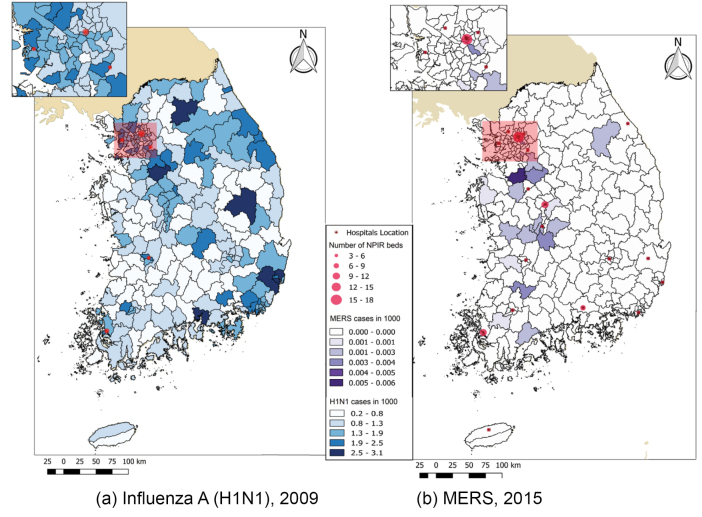
A Mismatch Map of the Ratio of Influenza A (H1N1), MERS, and NPIR Distribution. Abbreviations: NPIR, negative pressure isolation room; MERS-CoV, Middle East respiratory syndrome coronavirus.

Specifically, Figure 3a shows that NPIRs were not well allocated for the influenza A outbreak in 2009. The color gradation in the figure represents the ratio of confirmed influenza A patients to the population. It appears that more NPIRs were needed in the high prevalence areas, such as in the eastern and the southeastern areas, while NPIRs were concentrated in the west. Even in within the high-density Seoul area (enlarged in figure), NPIRs placement was not aligned with need, as shown in Figure 3a. After expanding NPIR capacity, the MERS-CoV outbreak occurred in 2015. Although it appears in Figure 3b that NPIRs were more evenly distributed compared to the past, there were still some areas with mismatches between the NPIR capacity and MERS-CoV cases. The central regions had insufficient facilities while the southern areas had excess facilities. As found in Figure 3b inset of the Seoul area, most MERS cases occurred in the south of the area, yet the NPIRs are primarily located north of the urban area.

This GIS mapping approach suggests a substantial mismatch of allocation of NPIRs, indicating that the Korean government should consider an evidence-based spatial method to allocate NPIRs funds based on a specific pattern of infectious disease and existing resource capacity. This study provides some evidence to be used to allocate the resources of health facilities based on population and case distribution and capacity of health facilities.

### Sensitivity Analysis With Two-Step Floating Catchment Area Method

**Figure 4:**
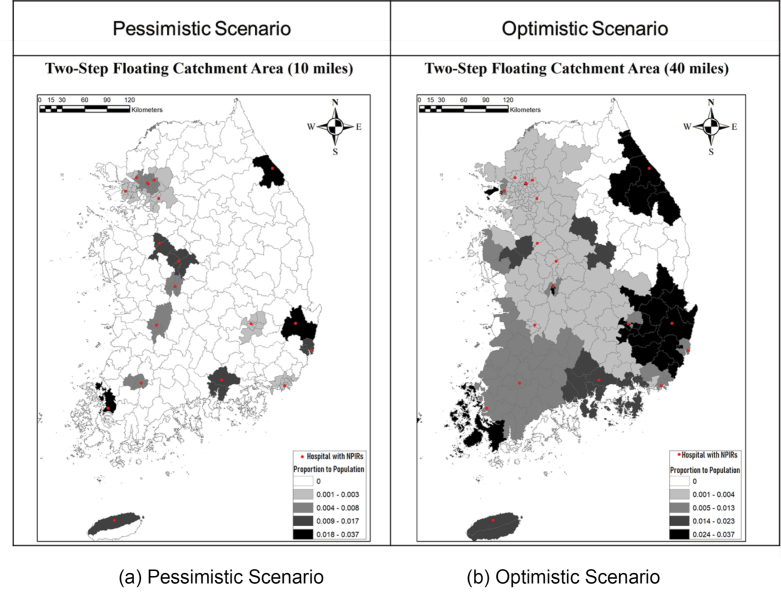
2SFCA Maps (Case 1. A Highly Contagious Disease Like COVID-19). Abbreviations: 2SFCA, two-step floating catchment area; COVID-19, coronavirus disease 2019; NPIRs, negative pressure isolation rooms.

To show the results of 2SFCA analyses, two types of infectious diseases (a highly contagious disease like COVID-19 vs. a hospital-based transmission like MERS-CoV) were used with two scenarios based on a travel distance to NPIR hospitals. Figure 4 shows the 2SFCA maps for the outbreak of a highly contagious disease, assuming that the whole population has an equal probability of infection. Figure 4a indicates that most areas in the country are not covered by the NPIR buffers of 10 miles. Even in the optimistic scenario shown in Figure 4b, which uses 40 miles driving distance, uncovered areas still exist in the east. We also note the magnitude of coefficients of proportions. Most areas have coefficients of less than 0.013, which implies that only 13 of the 1 million people in the region can be covered effectively. The analysis highlights the massive shortage of NPIRs for this type of disease.

**Figure 5:**
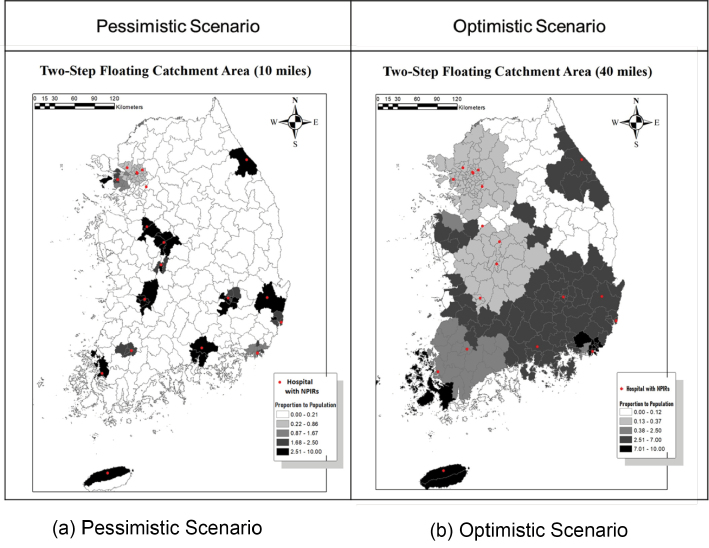
2SFCA Maps (Case 2: A Hospital-Based Transmission Like MERS-CoV). Abbreviations: 2SFCA, two-step floating catchment area; MERS-CoV, Middle East respiratory syndrome coronavirus; NPIRs, negative pressure isolation rooms.

Concerning another type of outbreak of a less contagious (but high mortality) disease like MERS, similar patterns of NPIR facility coverage are found in Figure 5. In Figure 5a, even though the magnitudes of ratio coefficient are greater than the first case, there are still areas without access to NPIR facilities. Moreover, the Seoul metropolitan area is the only place to be well covered by the NPIRs hospitals. The magnitude of coefficients of 2SFCA is also concerning. In the case of MERS in 2015, only 0.01~0.04% of regional populations could receive benefits from the current NPIR facilities. Thus, it may well be that most people would not have access to NPIRs even within the Seoul Metropolitan area, if there were a dense concentration of cases. In the optimistic scenario with 40-mile buffers shown in Figure 5b, while NPIR locations better cover the country, there still are some areas without coverage in the central southern region and the west and east coasts. Moreover, a longer distance assumption for service implies contagion risk of diseases while transferring patients. In additions, the coefficient of is not big enough to cover high density areas in South Korea either, so more facilities should be allocated in the areas.

**Figure 6:**
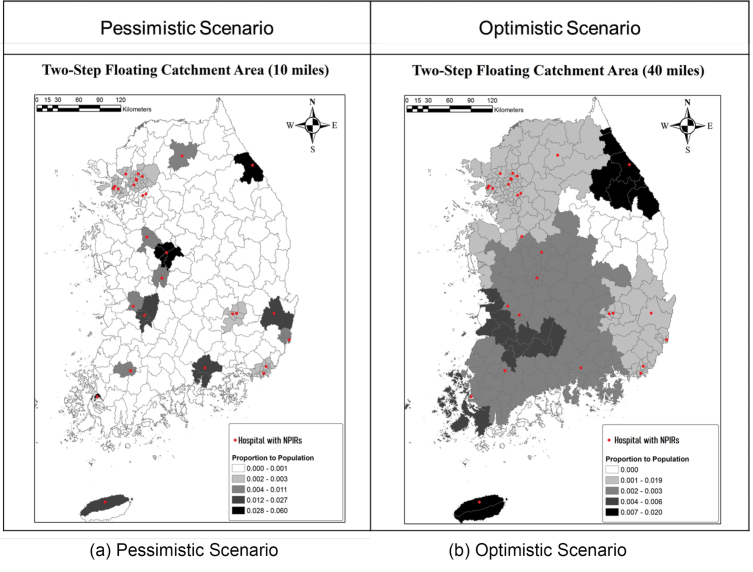
Two-step Floating Catchment Maps With Updated Data in 2018. Abbreviation: NPIRs, negative pressure isolation rooms.

Since the MERS outbreak, South Korean government has increased the number of NPIRs. As of 2018, there were 163 NPIRs installed in 29 hospitals across the country. We tested the allocation of NPIRs with the 2018 population in the context of a highly contagious disease in which the entire population has equal possibility of exposure. As shown in Figure 6, the overall spatial coverage expanded, but the magnitude of coefficients of NPIRs per capita coverage did not sufficiently increase compared to past allocation, particularly in a pessimistic scenario (Figure 6a). This finding indicates that there should still be more NPIRs across the country to be able to respond effectively to highly contagious disease like COVID-19. New NPIR resources need to be allocated in regions where there is currently low accessibility to NPIRs. Due to the difficulty in predicting disease spread patterns and the scope of risk, adequate funding and resources may need to be allocated to provide sufficient number of NPIRs in readiness for pessimistic scenarios.^40^

## Discussion and Conclusion

This study finds that NPIR allocation in South Korea is both suboptimal and inadequate for infectious disease risk, something that is particularly concerning in light of novel outbreaks such as COVID-19. In 2003, as a reaction to South Asian countries suffering from SARS, the Korean government established a strategic plan for infectious disease. However, despite the severity of the SARS pandemic, the government did not complete the plan until experiencing the severe infectious disease outbreak of MERS in 2015. During the period between the H1N1 and MERS outbreaks, the national plan for building NPIRs was not fully implemented, due to budgetary constraints and a lack of political interest. This issue remains unresolved during current COVID-19 pandemic.

This study reveals that South Korea lacks the requisite number of NPIR beds required to respond to disease outbreaks. There are three aspects to this shortage. First, based on the analysis of past cases, the absolute number of NPIRs is likely to be insufficient to care for all confirmed cases in an epidemic. Secondly, the allocation of NPIR resources to hospitals has not matched the regional ratio of patients to population in past pandemics. Finally, our analysis discloses significant and intensifying mismatching of NPIRs to confirmed cases of contagious diseases at the national level. Our conclusions strongly support the position that South Korea should conduct evidence-based allocation rather than be driven by crises of current epidemics and other political scenarios. To set criteria for a more effective allocation of NPIRs, systematic mechanisms for optimizing coverage should be developed and adopted by the national authorities.

Of course, evidence-based policy-making will not be a panacea and may not appear feasible in the midst of rapidly evolving threats such as COVID-19, but a systematic and scientific process will lead to better public health outcomes. The COVID-19 pandemic has revealed the many unanticipated or uncontrollable challenges which occur in responding to a novel and widespread disease. During a crisis, all types of systems, including physical, social, technical, and political will be tested. What our analysis suggests is that there are tangible preparations that can be made, in spite of the unknowns. One of these is infrastructure, namely the NPIRs, which are often costly and seemingly underutilized during normal times, but are essential during emergencies. Such facilities, like public goods generally, will be underprovided without good leadership and management during normal planning processes, rather than during emergencies. While we must respond to crises as best as our institutions are able, we also need to learn and strengthen our institutions beyond one crisis and before another.

Although we argue for the value of a simple spatial analytical approach which could be easily applied in situations of limited resources and data, this study has limitations in that is not designed to predict the occurrence of cases of highly contagious diseases.^13^ The assumption of using the population distribution as a proxy for potential risk groups may not be appropriate or sufficient for predicting demand of NPIRs in response to outbreaks. Socio-economic factors in local communities such as mobility, a level of social development, and types of social interactions need to be considered in analytic models for better prediction. In addition, our estimation of distribution for future outbreaks assumes uniform access to the nearest NPIR health facilities when using the 2SFCA model, which may fail to incorporate other characteristics of access such as forms of transportation. The limitations, however, do not undermine our conclusion of the need for more and better distributed facilities. For country-level planning, a simple approach suggests substantial need and suboptimal allocation which should be better addressed in the policy-making process. However, future studies should incorporate all of these factors into more advanced methods, such as an enhanced 2SFCA^34^ or multi-modal 2SFCA.^35^

## Ethical issues

There is no ethical issues. All data in this paper is publicly available.

## Competing interests

Authors declare that they have no competing interests.

## Authors’ contributions

DK and CKL developed the research idea and supervised the study. HK obtained the data and conducted the data analyses. HK drafted the initial manuscript and DK and CP critically reviewed and revised the manuscript. All authors reviewed and approved the final manuscript.

## Authors’ affiliations

^1^Institute of Public Policy and Administration, Chung-Ang University, Seoul, South Korea. ^2^School of Economic, Political and Policy Sciences, University of Texas at Dallas, Richardson TX, USA. ^3^Department of Public Administration, North Carolina Central University, Durham, NC, USA. ^4^Department of Urban Policy and Administration, Incheon National University, Incheon, South Korea.

## Funding

This work was supported by Incheon National University International Cooperative Research Grant in 2017.

## Key messages

**Implications for policy makers**

- In an infectious disease outbreak, government agencies should prepare sufficient numbers of infection control facilities in hospitals, such as negative pressure isolation rooms (NPIRs), to reduces airborne disease transmission.
- Allocating resources for NPIRs should be based on evidence on spatial distribution of disease risks and demand for the facility, rather than administrative convenience or political dynamics.
- NPIR allocation in South Korea is both suboptimal and inadequate for the infectious disease risk, which is particularly concerning in light of novel outbreaks such as coronavirus disease 2019 (COVID-19).
- To set criteria for a more effective allocation of NPIRs, systematic and scientific mechanisms for distributing resources and optimizing coverage should be developed and adopted by the national authorities for better public health outcomes.
- Even a simple method of spatial allocation (ie, two-step floating catchment area, 2SFCA) can be used to discover the lack of NPIR facilities and the locations where new NPIRs need to be established.

**Implications for the public**

The increasing emergence of virulent infectious diseases is cause for concern, as is evidenced in the coronavirus disease 2019 (COVID-19) outbreak originating in Wuhan, China and leading to a global pandemic in 2020. Despite the importance of negative pressure isolation rooms (NPIRs) in preventing the transmission of airborne infection, NPIR allocation in South Korea remains insufficient. A national plan for building NPIRs was not completed due to budgetary constraints and a lack of political interest, even after a series of outbreaks. South Korea needs to have more NPIRs and allocate them more wisely in order to respond to novel infectious disease outbreaks such as COVID-19. Citizens should ensure that the government conducts evidence-based allocation of NPIRs and other health facilities, rather than be driven by administrative convenience or other political factors.
